# Preparation, Characterization, and Wound Healing Promotion of Hydrogels Containing Glucosyloxybenzyl 2-Isobutylmalates Extract from *Bletilla striata* (Thunb.) Reichb.f.

**DOI:** 10.3390/ijms251910563

**Published:** 2024-09-30

**Authors:** Fei Ran, Kailang Mu, Gang Liu, Yuchen Liu, Yuxin Pang, Guo Feng, Lingli Zhou, Leqiang Peng

**Affiliations:** College of Pharmacy, Guizhou University of Traditional Chinese Medicine, Guiyang 550025, China; rf991125@163.com (F.R.); mukailang1@163.com (K.M.); pyxmarx@126.com (Y.P.); 13885184463@163.com (G.F.); zhoulingli0220@163.com (L.Z.); penglq2208@163.com (L.P.)

**Keywords:** glucosyloxybenzyl 2-isobutylmalates, *Bletilla striata* (Thunb) Reichb.f., plant materials, hydrogel, wound healing

## Abstract

Plant-derived medicinal materials have significant potential and promising applications in wound healing and skin regeneration. This study aims to develop a plant-based extract hydrogel from *Bletilla striata* (Thunb.Reichb.f.), specifically a glucosyloxybenzyl 2-isobutylmalates extract (B), and characterize its potential effects on wound healing. We synthesized the hydrogel using carbomer (C), glycerol (G), and triethanolamine (T) as the matrix, incorporating B into the hydrogel base, and evaluated its physical and chemical properties. In vitro tests assessed the biocompatibility of the glucosyloxybenzyl 2-isobutylmalates-carbomer-glycerol-triethanolamine (B-CGT) hydrogel and its effects on cell proliferation, migration, and adhesion. Animal model experiments evaluated its potential to promote wound healing. The results showed that the prepared B-CGT hydrogel possessed a good three-dimensional network structure and stability, demonstrating significant free radical scavenging capacity in antioxidant tests. In cell experiments, the B-CGT hydrogel exhibited no potential cytotoxicity and showed good hemocompatibility and promotion of cell proliferation. Animal experiments indicated that wounds treated with the B-CGT hydrogel healed significantly faster, with improved formation of new epithelial tissue and collagen. This study suggests that the developed B-CGT hydrogel is a promising candidate for wound dressings, with excellent physicochemical properties and controlled drug release capabilities, effectively promoting the wound healing process.

## 1. Introduction

Wound management is a crucial area in modern medical practice, with the research and development of wound treatment materials being an active field, particularly in finding effective treatment strategies for acute and chronic wounds [1,2,3,4]. Wound healing is a complex biological process involving a series of interacting cells, cytokines, and biomolecules [5]. The fundamental stages of this process include hemostasis, inflammation, tissue proliferation, and tissue remodeling. During this process, appropriate wound dressings can provide a moist environment, prevent infection, and promote cell migration and neovascularization, thereby accelerating the healing process [6].

Natural plant-derived medicinal materials hold significant potential and application prospects for wound healing and skin regeneration due to their good biocompatibility, biodegradability, low toxicity, antibacterial properties, and multifunctionality [7,8,9,10,11]. The medicinal plant *Bletilla striata* (Thunb) Reichb.f., known for promoting wound healing, has been used in China for over 2000 years [12,13,14]. Over the past decades, research has identified key bioactive compounds, such as polysaccharides from *B. striata*, polyphenols, and glucosyloxybenzyl 2-isobutylmalate, which have been shown to promote fibroblast proliferation, accelerate collagen synthesis, and exhibit anti-inflammatory and antioxidant properties [15,16,17,18]. However, the clinical application of *B. striata* polysaccharides and polyphenols is limited by challenges in optimizing extraction processes and ensuring consistent efficacy across different formulations. Additionally, *B. striata* polysaccharides face significant challenges in drug dispersion systems due to their high viscosity and difficulties in purification [15,16,19,20]. In contrast, glucosyloxybenzyl 2-isobutylmalate not only demonstrates excellent biological activity but also exhibits strong stability during extraction processes, allowing for more effective retention of the primary active components of *B. striata*, thus presenting potential value for further development and utilization [21,22,23].

Recent studies indicate that colloidal-system-based controlled release technology shows significant potential in drug delivery [24,25,26]. Hydrogels, noted for their high water content, excellent biocompatibility, and ability to mimic the extracellular matrix (ECM), have garnered considerable attention as materials for this purpose [27,28]. Moreover, hydrogels provide an ideal, moist environment for wounds, aiding in moisture retention, cellular migration, proliferation, and neovascularization [29]. Their favorable biocompatibility and biodegradability can minimize tissue irritation and side effects [30]. In recent years, numerous studies have focused on developing novel hydrogels with antibacterial, anti-inflammatory, and wound-healing properties to enhance healing outcomes [31,32]. For instance, hydrogels composed of sodium alginate and gelatin can effectively control the drug release rate, thereby improving therapeutic efficacy. This approach not only enhances drug bioavailability but also prolongs its duration of action in the body [33,34].

This study aims to develop a novel hydrogel derived from *B. striata* extracts of glucosyloxybenzyl 2-isobutylmalate and to explore its potential applications in promoting wound healing. Initially, the extraction and isolation techniques optimized by our research team were employed to enrich the glucosyloxybenzyl 2-isobutylmalate from *B. striata*. Furthermore, in vitro cellular assays and animal wound models were utilized to evaluate the wound healing efficacy of the developed hydrogel. Specifically, we monitored the effects of the hydrogel on cell migration, proliferation, and differentiation, assessing its influence on fibroblasts and other cell types closely associated with wound healing. Additionally, the effectiveness and biocompatibility of the hydrogel were evaluated through animal wound models. Notably, we focused on the hydrogel’s regulatory effects on inflammatory responses, its role in angiogenesis, and its promotion of collagen synthesis, thereby providing a comprehensive assessment of its application potential in wound healing. We anticipate that the hydrogel developed from *B. striata* extracts of glucosyloxybenzyl 2-isobutylmalate will integrate these advanced technologies, further enhancing its value in chronic wound healing applications.

## 2. Results and Discussion

### 2.1. Preparation of Hydrogel

#### 2.1.1. Carbomer-940 Is More Suitable as a Hydrogel Matrix for B-CGT

**Figure 1:**
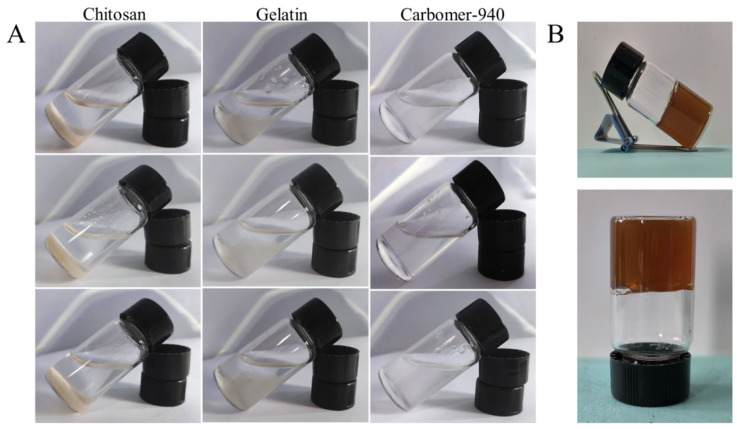
Hydrogel matrix screening. (**A**) Swelling effect of chitosan, gelatin, and carbomer in three different hydrogel matrices, each tested in parallel through three tests. (**B**) Represents the drug-loaded hydrogel picture when carbomer is used as the matrix.

The gel matrix can be either aqueous or oil-based [35,36]. However, based on the properties of glucosyloxybenzyl 2-isobutylmalates-carbomer-glycerol-triethanolamine (B-CGT), it is more suitable for preparation as an aqueous hydrogel. Aqueous gel matrices typically consist of water, propylene glycol, or glycerol combined with carbomers, alginates, gelatin, cellulose derivatives, or chitosan. Based on a literature review and preliminary experiments, chitosan, gelatin, and carbomer were chosen as candidate matrices for this study [37]. As shown in Figure 1A and Table 1, carbomer-940 was selected as the gel matrix after comprehensive evaluation. The carbomer-940 gel appeared as a white semi-solid with a smooth and transparent texture, minimal bubbles, good fluidity, and suitable viscosity.

Carbomer itself is a hydrophilic polymer that forms a three-dimensional network structure with high water content resembling an extracellular matrix (ECM), making it an excellent material for topical drug delivery [38]. The overall scoring, considering gel appearance, fluidity, and viscosity, indicated that carbomer-940 in concentrations ranging from 0.3% to 0.9% prepared using method I resulted in the best blank gel. This gel was water-soluble, odorless, smooth, and delicate, with strong adhesion and prolonged skin retention of the drug. It is easy to clean without a greasy feel, providing a comfortable application (Table 2).

Thus, carbomer-940 was selected as the gel matrix due to its white semi-solid appearance, moderate viscosity, good formability, and stability without layering. Concentrations of 0.3%, 0.6%, and 0.9% of carbomer-940 were used as the matrix for drug incorporation and mixing experiments. The hydrogel containing B-CGT was a brownish-yellow semi-solid with a uniform and delicate texture. It was easy to apply and more suitable for preparation as an aqueous gel formulation (Figure 1B).

#### 2.1.2. Screening of Moisturizers and pH Regulators

The selection of humectant dosage was investigated, with the results shown in Table 3. It was found that 15% and 20% glycerol resulted in an overly greasy product. Without glycerol, the hydrogel lacked moisturizing properties. At 5% glycerol, the moisturizing effect was inadequate. When glycerol was at 10%, the hydrogel appeared as a brownish-yellow semi-solid with good spreadability, moderate viscosity, and good stability. Additionally, it provided a comfortable cooling sensation when applied to the skin.

For pH regulator selection, the results are shown in Table 4. When 0.6 mL of triethanolamine was added to a gel containing 0.6% carbomer-940 and 10 mL of glycerol diluted to 100 mL with water, the pH was 5.62, which is close to the pH range of human skin (5.0–6.5) [39,40,41]. This formulation also appeared as a brownish-yellow semi-solid with good spreadability, moderate viscosity, no stratification, and good stability.

#### 2.1.3. Investigation of the Quantities of Various Additives in Hydrogels

**Figure 2:**
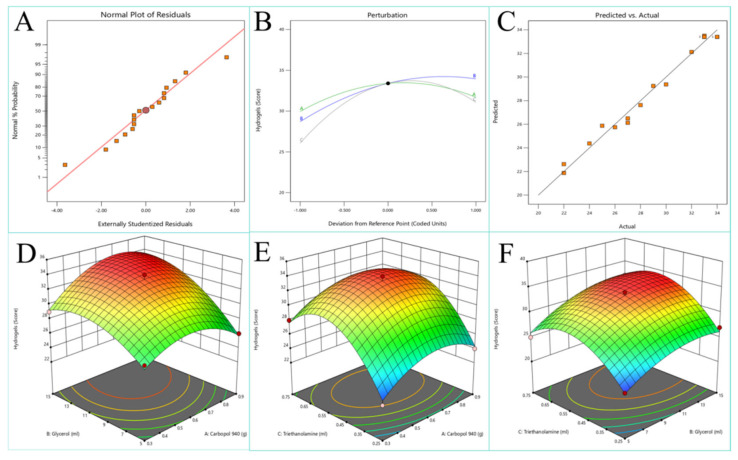
Response surface design results. (**A**) Normal probability distribution of the response value of the experimental residual. (**B**) Perturbation of each factor. (**C**) Comparison between actual values and predicted values. (**D**) Response surface plots for A and B factors. (**E**) Response surface plots for A and C factors. (**F**) Response surface plots for B and C factors.

The optimal quantities of various excipients in the hydrogel were determined using an orthogonal experimental design (Table 5). The experimental results are presented in Table 6. Data analysis was conducted using Design-Expert 13 software to produce response surface contour plots and 3D surface graphs, as shown in Figure 2. The fitted equation obtained from the data analysis is

(1) $$Y=33.40+0.8750A+2.63B+2.50C+1.25AB+0AC+0.5BC-2.58A^{2}-2.08B^{2}-4.825C^{2}$$

**Figure 3:**
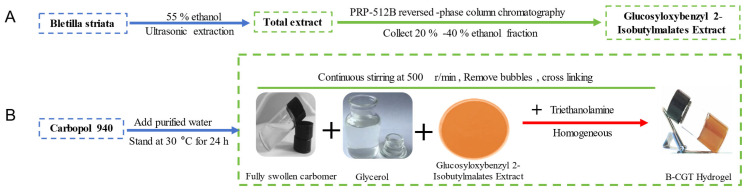
Schematic diagram of drug extraction and hydrogel synthesis route. (**A**) Extraction process of glucosyloxybenzyl 2-Isobutylmalates from Bletilla striata. (**B**) A schematic diagram of the synthesis path of B-CGT hydrogel.

The optimized formulation consisted of 0.71% Carbopol 940, 13.89% glycerol, and 0.57% triethanolamine. The hydrogel prepared with the optimized formulation was a uniform, stable, brownish-yellow semi-solid with good adhesiveness. The preparation process of B-CGT hydrogel is shown in Figure 3A,B.

### 2.2. Appearance and Centrifugal Stability

The appearance of the hydrogel was inspected and found to comply with the requirements of the 2020 edition of the Chinese Pharmacopoeia (Volume IV, General Principle 0114 on Gels) [42]. The hydrogel was uniform and delicate, and it maintained its gel-like state at room temperature without drying or liquefying, demonstrating good stability, as shown in Figure 1B.

**Figure 4:**
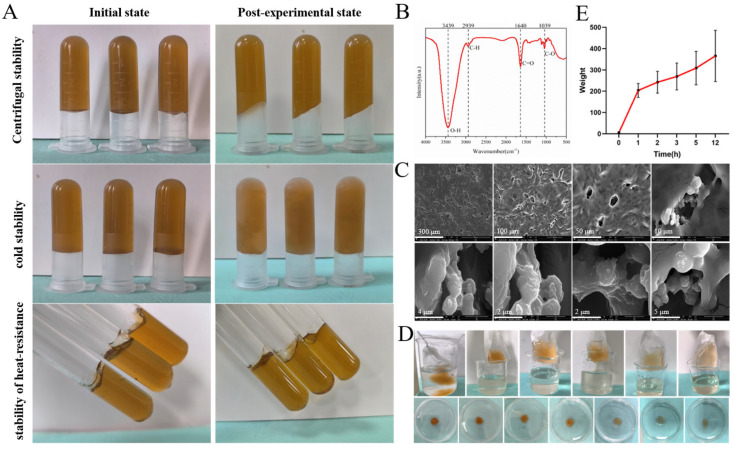
Physical properties and characterization of B-CGT hydrogels. (**A**) Centrifugal stability, heat stability, and cold stability tests were carried out under different conditions to evaluate the stability of B-CGT hydrogels under different conditions. (**B**) Fourier transform infrared spectroscopy (FTIR) analysis showed the characteristic peaks corresponding to the functional groups in B-CGT hydrogels. (**C**) SEM images showed the porous network structure of the hydrogel after freeze-drying, and the pore size ranged from 2 to 300 μm. (**D**) The expansion test shows the hydration capacity within 12 h, and the maximum expansion rate is 203.71%. (**E**) The swelling behavior of the B-CGT hydrogel over 12 h. The swelling ratio of the hydrogel increases significantly over time, with the most prominent swelling rate observed during the first hour.

The hydrogel exhibited good centrifugal stability, effectively eliminating air bubbles. The thermal stability and freeze–thaw stability at different temperatures and humidity levels were assessed to evaluate the storage conditions of the formulation. These studies provided a theoretical basis for determining the subsequent transportation and storage conditions of the drug. As shown in Figure 4A, the hydrogel retained its morphology without any layering, drying, or liquefaction, maintaining good uniformity in accordance with the 2020 edition of the Chinese Pharmacopoeia (Volume IV, General Principle 0114 on Gels).

### 2.3. Infrared and Morphological Studies

The Fourier transform infrared (FTIR) spectrum of the B-CGT hydrogel is presented in Figure 4B. In the FTIR spectrum of the B-CGT hydrogel, the strong absorption band around 3300–500 cm^−1^ is associated with the O-H bond stretching vibration, which is commonly observed in hydrogels due to the abundance of hydroxyl groups, indicating the presence of alcohols or phenols [43]. The peak near 2900 cm^−1^ is typically attributed to the C-H bond stretching vibration, suggesting the presence of alkanes [44]. The sharp peak around 1700 cm^−1^ is related to the C=O bond stretching vibration, indicative of ketones, aldehydes, or carboxylic acids. The peak at 1066 cm^−1^ signifies the C-O bond stretching vibration [45]. These spectral characteristics correspond to the chemical bond features of B-class components in *B. striata* [46].

After freeze-drying the hydrogel, its internal structure was observed using Scanning Electron Microscopy (SEM), as shown in Figure 4C. The hydrogel exhibited a loose and porous internal structure with irregular microcavities. These microcavities, possessing a three-dimensional network structure, can absorb and retain a significant amount of water, providing a moist environment for wounds and preventing secondary damage. Additionally, this structure effectively mimics the extracellular matrix (ECM), offering support and guidance for cells [47,48]. The three-dimensional network also imparts mechanical strength, providing physical protection to the wound. Furthermore, it can efficiently absorb wound exudate and lock it within the network, preventing leakage, reducing bacterial growth environments, lowering the risk of infection, and promoting wound healing.

### 2.4. Swelling Properties

Figure 4D presents the real image of the hydrogel swelling due to hydration. The hydrogel visibly swells and increases in volume as it absorbs water, without dissolving after 12 h. As shown in Figure 4E, the swelling rate of the hydrogel significantly increases over time, initially rising rapidly to 203.71% within the first hour and then gradually stabilizing between 10 and 12 h, demonstrating excellent water absorption capacity. This indicates the hydrogel’s ability to absorb large amounts of liquid over a period, thus effectively managing wound exudate, cleaning the wound promptly, reducing the risk of bacterial infection, and accelerating wound healing. Additionally, the soft and moist hydrogel provides a humid environment for the wound, preventing secondary trauma caused by dressing changes [49,50].

### 2.5. Oxidation Resistance

To evaluate the antioxidant performance of the hydrogel, this study employed both DPPH and ABTS methods to measure its free radical scavenging capacity. The results indicated that the hydrogel exhibited a free radical scavenging rate of 69.28 ± 1.31% using the DPPH method, demonstrating strong antioxidant capacity. Further testing using the ABTS method revealed a free radical scavenging rate of 62.82 ± 12.73%. Both methods confirmed that the prepared hydrogel possesses significant antioxidant properties, potentially reducing oxidative stress during wound healing [51,52].

### 2.6. The B-CGT Hydrogel Has Good Biocompatibility

#### 2.6.1. Cellular Compatibility

**Figure 5:**
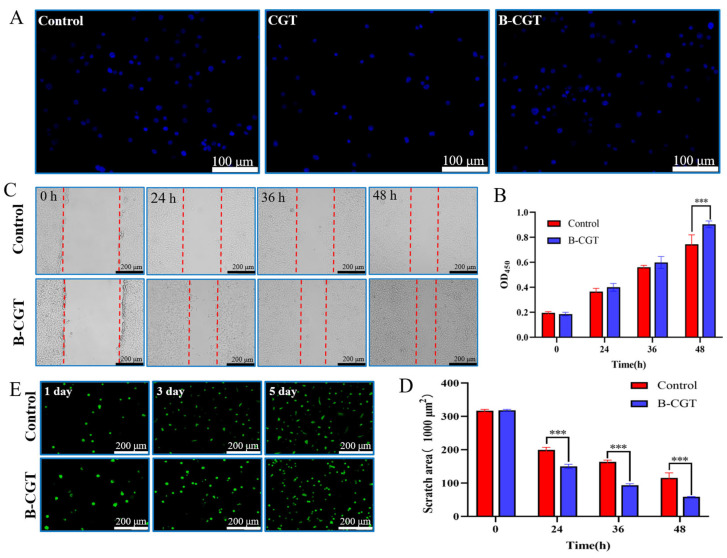
The cytocompatibility of B-CGT hydrogel. (**A**) Adhesion test of NIH/3T3 cells on B-CGT hydrogel. (**B**) Effect of B-CGT hydrogel on the proliferation of NIH/3T3 cells, *** *p* < 0.001. (**C**) Cell scratch assay. (**D**) Scratch area of NIH/3T3 cells under the intervention of B-CGT hydrogel; smaller scratch areas indicate higher migration rates, *** *p* < 0.001. (**E**) Toxicity test of B-CGT hydrogel on cells.

Cell adhesion is fundamental for communication between cells and their microenvironment, influencing migration, proliferation, differentiation, and other essential cellular behaviors. During wound healing, the adhesion and migration of cells are crucial [53,54]. As shown in Figure 5A, NIH/3T3 cells effectively adhered to CGT and B-CGT hydrogels after a 4 h incubation, displaying behavior similar to cells on TCP culture dishes. This indicates that CGT and B-CGT support fibroblast adhesion and distribution, although CGT had fewer attached cells compared to B-CGT.

The CCK-8 assay was used to quantitatively assess the impact of B-CGT hydrogel on NIH/3T3 cell proliferation. As shown in Figure 5B, cells cultured on B-CGT hydrogels exhibited significant proliferation after 24 h. After 48 h, cell numbers increased significantly, with the B-CGT group showing a higher OD value compared to the control group. In the cell migration assay (Figure 5C), cells in the B-CGT group migrated further than those in the control group (*p* < 0.05), a finding supported by the results in Figure 5D.

Live/dead staining was used to evaluate the cytotoxicity of B-CGT hydrogels. Figure 5E shows that NIH/3T3 cells had a survival rate above 76.74 ± 5.58% after 5 days of culture in both the B-CGT group and the control group (medium without gel). According to ISO 10993-5 standards, a survival rate above 70% indicates no potential cytotoxicity, which is suitable for further biocompatibility evaluation [55].

These results collectively demonstrate that B-CGT hydrogels exhibit excellent cellular compatibility, promoting cell proliferation, migration, and adhesion. B-CGT hydrogels provide a conducive environment for cell adhesion, spreading, and growth compared to the control group.

#### 2.6.2. Hemocompatibility

**Figure 6:**
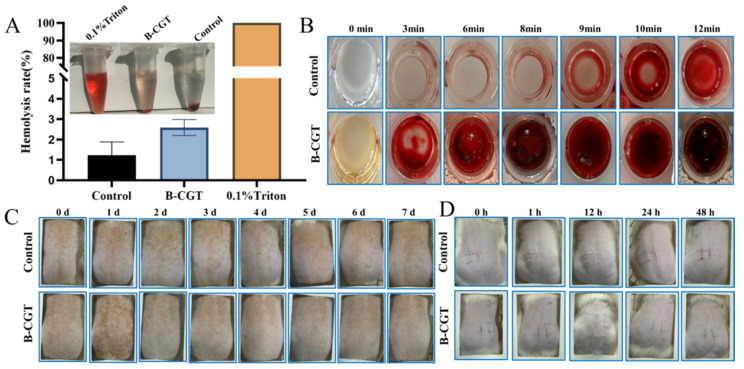
Blood compatibility and safety test. (**A**) Hemolysis test diagram and hemolysis rate statistics of B-CGT hydrogel. (**B**) The coagulation of the control group and the B-CGT hydrogel group at different time periods (0, 3, 6, 8, 9, 10, 12 min). (**C**) The skin irritation test was performed on rat skin to evaluate the erythema and edema of the control group and the B-CGT hydrogel group within 7 days. (**D**) The scratch stimulation test was performed on rats to evaluate the scratch transformation of the control group and the B-CGT hydrogel group within 48 h. The graph shows that there is no obvious inflammatory reaction in the B-CGT hydrogel group.

Hemolysis testing is crucial for assessing the biocompatibility and safety of materials. According to ISO 10993-4 standards, a hemolysis rate (HR) ≤ 5% is considered non-hemolytic [56,57]. B-CGT hydrogels exhibited an HR of less than 3% (2.48 ± 0.40%, n = 6), as shown in Figure 6A, indicating good hemocompatibility.

In wound healing, managing minor continuous bleeding post-debridement is critical. Effective hemostasis impacts the normal healing process. The coagulation time of blood treated with B-CGT hydrogel was measured to preliminarily evaluate its hemostatic capability. Figure 6B shows that while whole blood coagulated in 12 min, stable clots formed in about 3 min upon contact with the B-CGT hydrogel, demonstrating effective hemostasis compared to the control group.

The prepared B-CGT hydrogel exhibited excellent biocompatibility and significant promotion of wound healing, as demonstrated by the in vivo and in vitro experiments. Compared to other plant-derived hydrogels, our formulation shows several advantages. For instance, alginate-based hydrogels are widely used due to their moisture retention properties, but they often result in local acidification, which can delay the healing process. Similarly, hyaluronic-acid-based hydrogels provide excellent cell migration support but suffer from rapid degradation, requiring frequent replacement [31,58,59,60]. In contrast, the B-CGT hydrogel combines excellent moisture retention with longer durability and better mechanical strength, making it more suitable for long-term wound care applications (Table 7).

### 2.7. Safety Performance

Following ISO 10993-10 standards, the irritation potential of B-CGT hydrogels on skin was assessed [61]. Figure 6C shows no erythema or edema on the backs of mice in both the B-CGT and control groups. As seen in Table 8 (n = 4), the Primary Irritation Index (PII) for B-CGT was low, which is similar to the control group, indicating no irritation. Figure 6D shows the further evaluation of safety using the scratch test, revealing no erythema, edema, or eschar formation in the B-CGT group. The results in Table 9 (n = 4) confirm the absence of irritation, demonstrating that B-CGT hydrogels are safe and non-irritating.

### 2.8. B-CGT Hydrogel Can Significantly Promote Wound Healing in Rats

**Figure 7:**
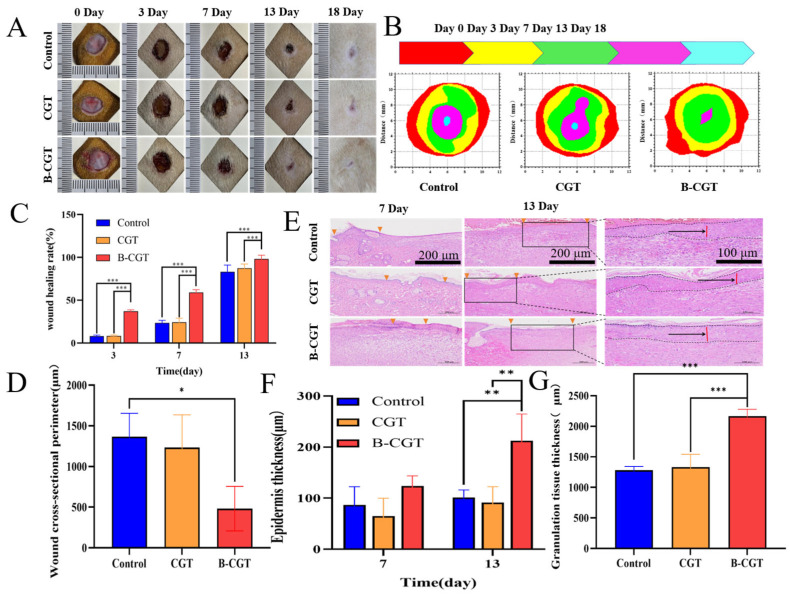
The effect of B-CGT on wound healing in rats. (**A**) The wound healing process of different groups of rats over different time periods (0, 3, 7, 13, 18 d). (**B**) The changes in the wound healing area in different groups of rats over different time periods (0, 3, 7, 13, 18 d). (**C**) Quantitative analysis of wound healing area in different groups of rats at different time periods (3, 7, 13 d). (**D**) The cross-sectional circumference of the wound on the 13th day of different groups of rats. (**E**) Wound tissue epithelial layer slice diagram of different groups of rats at 7 d and 13 d. (**F**) Epidermal thickness maps of different groups of rats at 7 d and 13 d. (**G**) The thickness of granulation tissue in different groups of rats on the 13th day; * *p* < 0.05, ** *p* < 0.01, *** *p* < 0.001.

To evaluate the in vivo wound healing efficacy of B-CGT hydrogel, an acute full-thickness wound model was established. As shown in Figure 7A, B-CGT hydrogel was applied to the wounds of SD rats, and wound healing was documented over time. Figure 7B illustrates significant changes in wound size across different groups at 0, 3, 7, 13, and 18 days. By day 3, B-CGT-treated wounds showed 37.09% healing, which was significantly higher than that of the CGT group (8.43 ± 0.03%) and the control group (8.05 ± 0.06%). By day 7, the B-CGT group exhibited a healing rate of 59.04 ± 0.07%, which was substantially higher than those of the CGT (24.47 ± 0.07%) and control groups (23.66 ± 0.17%). The CGT and control groups experienced prolonged inflammation and slower healing, with rates below 50%. Over time, the B-CGT hydrogel maintained superior healing. By day 13, the CGT and control groups showed about 80% healing, while the B-CGT group achieved over 90% (98.22 ± 0.02%), which was significantly higher than the control group (*p* < 0.05) (Figure 7C). The histological analysis in Figure 7D corroborates these results.

Re-epithelialization, a critical step in skin wound healing, precedes dermal repair. It quickly restores the wound’s functional barrier, preventing excessive transdermal water loss and infection risk [62]. To further assess the hydrogel’s effects on re-epithelialization and granulation tissue formation, observations were made on days 7 and 13. Figure 7E shows a relatively complete epithelial layer in the B-CGT group’s wound sections. In contrast, the CGT and NS groups displayed incomplete epithelial layers. On day 7, the NS group’s wounds remained open. By day 13, as shown in Figure 7F, the B-CGT group exhibited more pronounced re-epithelialization compared to the controls.

Granulation tissue growth, which is crucial for wound regeneration, provides structural support, fills wound cavities, and offers a matrix for epidermal cell migration [63]. It also ensures nutrient supply and waste removal through angiogenesis [64]. Granulation tissue thickness is an important indicator of healing efficacy [65]. As shown in Figure 7G, the B-CGT group’s granulation tissue thickened significantly more than the control group’s by day 13, indicating enhanced wound repair.

Overall, the full-thickness wound healing process involves a complex biological interplay between various phases and cell types. Epidermal and granulation tissues interact to facilitate wound closure. The epidermis provides coverage and protection, while granulation tissue offers support and nourishment. The coordinated development and remodeling of these tissues are key to successful wound healing and the restoration of normal skin function.

#### 2.8.1. B-CGT Can Effectively Promote the Transformation from the Inflammatory Phase to the Proliferative Phase

**Figure 8:**
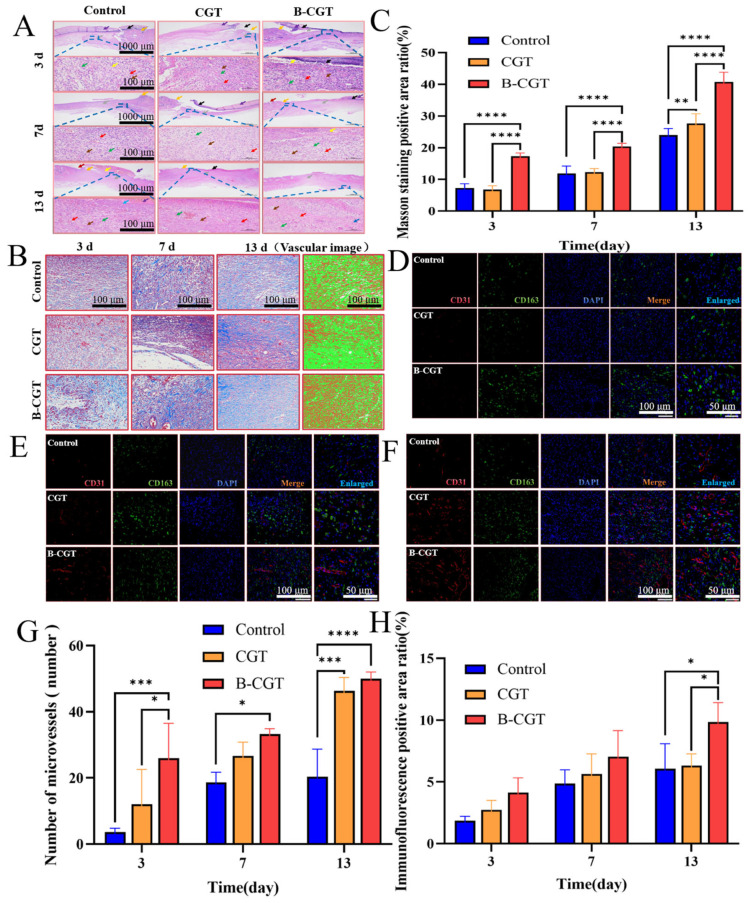
The effect of B-CGT on wound healing. (**A**) The morphological changes of the skin layer at different time points. (**B**) Masson staining analysis. (**C**) Masson positive area ratio. (**D**) Fluorescence double-labeled detection of wound tissue on the third day. (**E**) Fluorescent double-labeled detection of wound tissue on the seventh day. (**F**) Fluorescence double-labeled detection of wound tissue on the 13th day. (**G**) The number of microvessels in the wound tissue at different time points. (**H**) Immunofluorescence-positive area ratio. * *p* < 0.05, ** *p* < 0.01, *** *p* < 0.001, **** *p* < 0.0001.

Inflammation significantly impacts wound healing, with the epidermis and granulation tissue providing nourishment and numerous lymphocytes regulating immune responses to identify and eliminate pathogens. HE staining was used to observe morphological changes at different time points during wound healing [27,66,67,68]. Figure 8A shows that on day 3 post-treatment, all groups exhibited an incomplete epidermis and significant dermal necrosis (black arrows), with numerous necrotic cell debris and unstructured eosinophilic material, along with abundant inflammatory exudate (purple arrows). The edges of the injury showed epidermal thickening (orange arrows), some granulation tissue (gray arrows), new blood vessels (green arrows), fibroblasts (brown arrows), and numerous lymphocytes (red arrows), with minor bleeding (yellow arrows). By day 7, the control and CGT groups showed thin skin tissue with abundant inflammatory exudate. The CGT group exhibited extensive necrosis in the epidermis, with minimal granulation tissue proliferation. In contrast, the B-CGT group showed extensive granulation tissue proliferation, containing numerous fibroblasts, new blood vessels, and lymphocytes. On day 13, all groups showed further skin structure recovery, with the B-CGT group displaying a relatively complete epidermal structure and significant granulation tissue proliferation rich in collagen fibers and new blood vessels. The control and CGT groups showed slower recovery, with some hyperkeratosis in the epidermis (deep red color). Overall, B-CGT effectively promoted the transition from the inflammatory phase to the proliferative phase, accelerating wound healing.

#### 2.8.2. B-CGT Can Promote the Formation of Collagen Fibers

Increased collagen fibers in healing tissue are a hallmark of wound healing. Masson staining was used to observe collagen fiber distribution in the wound area [69,70]. Collagen fibers (blue), muscle fibers (red), and cell nuclei (black) were identified. On day 3 post-treatment, the control group showed minimal muscle fiber generation (red), with loose tissue structure and slight fibrosis (Figure 8B). The CGT and B-CGT groups showed more collagen fibers with signs of fibrosis. Compared to the control and B-H matrix groups, the B-CGT group had a denser tissue structure with visible initial blood vessel generation. By day 7, collagen fiber generation in the B-CGT group significantly increased, with denser tissue providing structural support and aiding repair and regeneration [71,72,73]. Both the CGT and B-CGT groups showed significant fibrosis with increased blood vessel formation, although it was not prominent. By day 13, the B-CGT group was most notable, with abundant collagen fibers, dense tissue, and significant tissue remodeling with increased blood vessels. As shown in Figure 8C, the positive area ratio in the B-CGT group was higher at all time points compared to the control and CGT groups. By day 7, the CGT group had a slightly higher positive area ratio than the B-CGT group. Overall, the B-CGT group consistently showed higher positive area ratios, demonstrating significant wound healing effects, likely through collagen fiber generation.

#### 2.8.3. B-CGT Can Promote the Expression and Distribution of CD31 and CD163 in Wound Tissue

Fluorescent double-labeling was employed to detect the expression and distribution of CD31 and CD163 in wound tissues. Concurrently, this study observed the microvascular count of CD31 (red fluorescence), the positive area ratio of CD163 (green fluorescence) (expression range), the average optical density (intensity of positive signals), and the area density (average intensity of positive signals within the measured tissue area), as well as the distribution of cell nuclei during the inflammatory, proliferative, and tissue remodeling phases [74,75].

As shown in Figure 8D, on the third day of tissue staining, both the control group and the CGT group exhibited almost no microvascular formation. In contrast, the B-CGT group showed a prominent red fluorescence, indicating that the B-CGT hydrogel promotes angiogenesis during the inflammatory phase. After seven days of treatment, Figure 8E reveals that the B-CGT group had significantly higher expressions of both vascular count (CD31) and green fluorescence (CD163) compared to the control and CGT groups, suggesting a positive trend in wound healing. This indicates that the B-CGT group developed more mature capillaries in the wound area.

Thirteen days post-treatment, the density of CD31 in the B-CGT group was significantly higher than that in the control group, as shown in Figure 8F. The control group had the poorest CD31 and CD163 expression and formation, while the B-CGT group demonstrated better expression of both markers. Overall, the wound healing process was slower in the control and CGT groups compared to the B-CGT group, which exhibited accelerated wound healing capabilities. Figure 8G illustrates the generation of microvessels at different time points, and Figure 8H shows that the positive expression of CD163 is consistent with these results (*p* < 0.001).

## 3. Materials and Methods

### 3.1. Materials

Chitosan (Batch No.: 2230614002), DPPH (Batch No.: 2230505001), and ABTS (Batch No.: 3230821001) were purchased from Beijing Solarbio Science & Technology Co., Ltd. (Beijing, China); gelatin (Batch No.: C15218933) and carbomer-940 (Batch No.: C15626960) were obtained from Shanghai Macklin Biochemical Co., Ltd. (Shanghai, China); glycerol (Batch No.: 23180341) was acquired from Landaco Technology Co., Ltd. (Beijing, China); triethanolamine (Batch No.: B2313574) and Methylparaben (Batch No.: A2125255) were sourced from Shanghai Aladdin Biochemical Technology Co., Ltd. (Shanghai, China); NIH/3T3 specialized culture medium (Batch No.: WHAA24C281) was procured from Wuhan Puno Saier Life Technology Co., Ltd. (Wuhan, China); PBS (Batch No.: GA24020023047), trypsin (Batch No.: GA2406091), DAPI staining reagent (Batch No.: CR2404001), the CCK-8 assay kit (Batch No.: CR2312062), the Calcein-AM/PI Live/Dead Cell Double Staining Kit (Batch No.: MPC2402034), Triton X-100 (Batch No.: CR2210021), saline (Batch No.: GA24010090444), eco-friendly dewaxing solution (Batch No.: G1128), general tissue fixative (Batch No.: G1101), the hematoxylin–eosin high-definition staining kit (Batch No.: G1076), iF488-Tyramide (Batch No.: G1231), CY3-Tyramide (Batch No.: G1223), iF647-Tyramide (Batch No.: G1232), FITC-Tyramide (Batch No.: G1222), citrate antigen retrieval solution (Batch No.: G1202), EDTA antigen retrieval solution (Batch No.: G1203), tissue autofluorescence quencher (Batch No.: G1221), bovine serum albumin (Batch No.: GC305010), DAPI staining reagent (Batch No.: G1012), and anti-fluorescence quenching mounting medium (Batch No.: G1401) were purchased from Wuhan Servicebio Technology Co., Ltd. (Wuhan, China). Isoflurane (Batch No.: 2024012501) was obtained from Shandong Anter Veterinary Technology Co., Ltd. (Shandong, China). Absolute ethanol (Batch No.: 100092683), xylene (Batch No.: 10023418), n-butanol (Batch No.: 100052190), and neutral balsam (Batch No.: 10004160) were acquired from Sinopharm Chemical Reagent Co., Ltd. (Shanghai, China).

### 3.2. Preparation of Glucosyloxybenzyl 2-Isobutylmalates Extracts of B. striata

Dried and pulverized *B. striata* material was added to 55% ethanol with a solid–liquid ratio of 1:10 g/mL. The mixture was subjected to ultrasonic extraction at a power of 344 W and a frequency of 40 kHz for three sessions of 30 min each. After each extraction session, the mixture was filtered to remove the residue. The collected filtrates were then concentrated under reduced pressure using a rotary evaporator until a thick extract was obtained.

This thick extract was prepared for further processing by mixing it with PRP-512B reversed-phase resin at a ratio of 1:15 (g/g). The resin was pre-treated by soaking it in 95% ethanol for 24 h, followed by packing it into a column. The packed column occupied approximately half of the column length. The column was then rinsed with 95% ethanol until the eluate mixed with water at a 1:5 ratio became clear. Further rinsing with water continued until no ethanol odor was detected.

The thick extract was then applied to the column using a dry loading method. Gradient elution was performed with water (until the eluate was colorless), 20% ethanol (until the eluate was colorless), and, finally, 40% ethanol (until the eluate was colorless). The fractions eluted with 40% ethanol were collected and concentrated under reduced pressure to obtain the final dry extract B, which was stored for further use [76].

### 3.3. Study of the Preparation Process of Hydrogel

#### 3.3.1. Selection of the Hydrogel Matrix

Three common excipients, Carbopol-940, chitosan, and gelatin, were chosen as potential hydrogel matrices. The preparation followed three different methods. (1) A suitable amount of hydrogel matrix was gradually sprinkled onto the water surface and left to swell for 12 h. (2) A suitable amount of hydrogel matrix was placed in a beaker, water was added gradually, and it was allowed to swell for 12 h. (3) Through a direct method, a suitable amount of hydrogel matrix was slowly added to water under continuous stirring at 1000 rpm for 4 h until no white substance was visible. The different matrices were then evaluated based on their form, texture, fluidity, and viscosity to preliminarily select the optimal hydrogel matrix. Specifically, Carbopol-940 was allowed to swell for 12 h and then stirred at 1000 rpm to remove air bubbles. Glycerin was added for cross-linking, triethanolamine was used to adjust the pH, and the volume was adjusted to 100 mL.

#### 3.3.2. Single-Factor Investigation

In single-factor experiments, each formulation used a total water volume of 100 mL, with component percentages given as g/100 mL.

1. Moisturizer Glycerin Selection

Blank Carbopol gel was prepared according to Section 3.3.1 with 1.0 g of the formula. When stirring, 1.25 g of B extract (glucosyloxybenzyl 2-isobutylmalates extract) and 0%, 5%, 10%, 15%, 20% glycerol were added, respectively. Then, 0.6 mL of triethanolamine was added, and the volume was adjusted to 100 mL with purified water to form the B-CGT self-adhesive hydrogel. The evaluation included appearance, viscosity, spreadability, and stability (under high temperature, low temperature, and centrifugation).

2. pH Adjuster Triethanolamine Selection

Blank Carbopol gel was prepared following the method in Section 3.3.1, and then 1.25 g of B extract and 10 mL of glycerin were added while stirring. Different amounts of triethanolamine were used to adjust the pH, and the volume was finally adjusted to 100 mL with purified water to form the B-CGT hydrogel. Evaluations included appearance, viscosity (a Kinexus rotary rheometer (Bonako (Changzhou) New Technology Co., Ltd., Changzhou, China) was used to determine the viscosity of the hydrogels), spreadability, stability (under high temperature, low temperature, and centrifugation), and pH.

#### 3.3.3. Response Surface Methodology for Hydrogel Formulation Optimization

Based on the single-factor experiments, the concentrations of Carbopol-940, glycerin, and triethanolamine were selected as the factors for further investigation. Using Design-Expert 13 software (Stat-Ease, Inc., Minneapolis, MN, USA) and a Box–Behnken design, a three-factor, three-level response surface methodology was employed to optimize the B-CGT hydrogel’s formulation. The sensory evaluation scores of the hydrogels were used as the response value to determine the optimal concentrations of the excipients. The design of the response surface methodology is shown in Table 10, and the scoring criteria are provided in Table 11.

### 3.4. Physical Properties Testing

The appearance of the gel was evaluated following the guidelines in Section 0114 of the Fourth Part of the Chinese Pharmacopoeia (2020 edition) [42]. A suitable pH level is beneficial for promoting wound healing. The pH of the semi-solid hydrogel was measured using a PHS-3C benchtop pH meter (Shanghai Chemical Laboratory Equipment Co., Ltd., Shanghai, China). The stability of the hydrogel, including its centrifugal stability, thermal stability, and cold stability, is crucial for ensuring its safety and effectiveness in practical applications. Centrifugal stability was evaluated through 80-1 electric centrifuge (Changzhou Runhua Electric Co., Ltd., Changzhou, China) at 3000 r/min for 30 min. The heat resistance stability of the DK-98-1 constant-temperature water bath pot (Tianjin Taisite Instrument Co., Ltd., Tianjin, China) was maintained in a 55 °C water bath for 6 h. The cold-resistant stability was tested at 4 °C for 24 h in the BCD-130EN Haier refrigerator (Qingdao Haier Refrigerator Co., Ltd., Qingdao, China). The swelling behavior of the B-CGT hydrogel was evaluated by examining its swelling ratio.

### 3.5. Characterization of the Hydrogel

The microstructure and the morphology of the hydrogel were observed using a TESCAN CLARA scanning electron microscope (SEM, Tesken (China) Co., Ltd., Shanghai, China). The testing environment involved rapidly freezing the hydrogel with liquid nitrogen to preserve a large amount of its water content. The hydrogel was then freeze-dried using a lyophilization method. A layer of conductive metal was coated on the surface of the freeze-dried hydrogel to reduce charge accumulation and enhance conductivity, and the sample was subsequently placed on the SEM stage for scanning and observation.

The chemical structure of the B-CGT hydrogel was characterized using a ThermoScientific Nicolet iS5 Fourier transform infrared spectrometer (FT-IR, Thermo Fisher (China) Scientific Inc., Shanghai, China) within the range of 4000 to 500 cm^−1^. This analysis enabled the investigation of the chemical bonds within the molecular network structure of the hydrogel.

### 3.6. Antioxidant Activity of Hydrogels (ROS Detection)

The freeze-dried hydrogels were extracted with PBS at a ratio of 1:50 for 24 h, and the extract was reserved for further use. The DPPH assay was employed to measure the antioxidant activity, with the working solution prepared according to the DPPH kit’s instructions. Absorbance was measured at 517 nm using a microplate reader (Thermo Fisher (China) Scientific Inc., Shanghai, China). The DPPH scavenging rate (%) was calculated as follows:

(2) $$\mathrm{DPPH}\;\mathrm{scavenging}\;\mathrm{rate}\;\left(\%\right)=\left(\frac{A_{1}-A_{2}}{A_{1}}\right)\times 100\%$$

where A_1_ represents the control group (water + DPPH solution) and A_2_ represents the hydrogel extract.

The ABTS method was used for further verification. The working solution was prepared according to the ABTS kit’s instructions. Absorbance was measured at 734 nm using a microplate reader. The ABTS scavenging rate (%) was calculated as follows:

(3) $$\mathrm{ABTS}\;\mathrm{scavenging}\;\mathrm{rate}\;\left(\%\right)=\left(\frac{A_{1}-A_{2}}{A_{1}}\right)\times 100\%$$

where A_1_ represents the control group (water + ABTS solution) and A_2_ represents the hydrogel extract.

### 3.7. Biocompatibility of Hydrogels

#### 3.7.1. Cell Compatibility of Hydrogels

To assess cell compatibility, B-CGT hydrogel was extracted with culture medium (1:30) for 24 h and then filtered using a 0.45 μm membrane. The cell proliferation capability was evaluated using the Cell Counting Kit-8 (CCK-8). NIH/3T3 cells were revived and cultured at 37 °C with 5% CO2. The cells were seeded in 96-well plates at a density of 2 × 10^3^ cells per well. After 4 h of incubation, the medium was replaced with 80 μL of extract solution and 20 μL of medium. At 24, 36, and 48 h, CCK-8 solution (10%) was added as per the kit instructions. After 2 h of incubation in the dark, the absorbance at 450 nm was measured using a microplate reader. The cell proliferation rate was calculated as

(4) $$\mathrm{Cell}\;\mathrm{Proliferation}=\frac{\left(OD_{a}-OD_{c}\right)}{\left(OD_{b}-OD_{c}\right)}$$

where OD_a_ represents the experimental group with B-CGT, OD_b_ represents the control group without cells, and OD_c_ represents the blank group without hydrogel and cells.

For the cell adhesion assessment, 3 × 10^5^ NIH/3T3 cells were seeded onto untreated 24-well plates under sterile conditions. Fifty microliters of B-CGT extract and CGT extract (non-drug-loaded hydrogel) were added to the wells and incubated for 4 h. A positive control was set by seeding an equal number of cells directly onto 24-well TCP culture plates. After 4 h, the medium was gently aspirated, and the cells were washed twice with PBS. The cells were stained with DAPI for 5 min and observed under a DMI 8 inverted microscope (Leica Microsystems GmbH, Heidelberg, Germany).

To investigate the effect of the hydrogel on fibroblast migration, a scratch assay was performed. NIH/3T3 cells (2 × 10^6^) were cultured in 6-well plates for 24 h. Scratches of uniform width were made at the bottom of each well, followed by the addition of hydrogel extract after removing the medium. The cells were incubated and observed under an inverted microscope at specific time points to monitor migration into the scratch area.

To evaluate cytotoxicity using live/dead cell staining, 1.5 × 10^4^ cells were seeded in 24-well plates. After incubation for 12, 24, and 36 h, the medium was removed, and 100 μL of live/dead staining solution was added to each well. Following 15 min of incubation in the dark, the staining solution was aspirated, the cells were washed twice with sterile PBS, and the cells were observed under an inverted fluorescence microscope at 1, 3, and 5 days.

#### 3.7.2. Blood Compatibility of Hydrogels

Blood compatibility was assessed via hemolysis tests. Fresh rat blood was centrifuged at 2000 rpm for 10 min to isolate RBCs, which were then washed three times with 0.9% NaCl to obtain purified RBCs. A 5% RBC suspension (*v*/*v*) was prepared. Then, 200 μL of RBC suspension was mixed with 800 μL of 0.9% NaCl (negative control), 200 μL of RBC suspension was mixed with 800 μL of 0.1% Triton X-100 (positive control), and 200 μL of RBC suspension was mixed with 100 μL of hydrogel extract and 700 μL of 0.9% NaCl (experimental group). After 2 h of incubation at 37 °C, the samples were centrifuged at 2000 rpm for 10 min, and photographs were taken. The supernatant was transferred to 96-well plates, and absorbance at 540 nm was measured using a microplate reader to calculate the hemolysis rate (HR).

(5) $$\mathrm{HR}\left(\%\right)=\left(\frac{A_{h}-A_{n}}{A_{t}-A_{n}}\right)\times 100\%$$

where A_h_, A_t_, and A_n_ represent the absorbance at 540 nm for the experimental, positive, and negative control groups, respectively.

#### 3.7.3. Blood Clotting Effect of Hydrogels

The blood clotting effect of the hydrogels was evaluated using an in vitro clotting test. Hydrogels were placed in 96-well plates and incubated in a water bath at 37 °C for 10 min to reach the desired experimental temperature. Subsequently, 45 μL of anticoagulated fresh rat blood was added, followed immediately by 9 μL of CaCl2 solution (25 mol/L). Blood clotting was observed and recorded at various time points. Unclotted blood was gently aspirated, and the wells were washed twice with 0.9% NaCl to remove non-clotted blood and impurities. The formation of clots was documented through photography.

### 3.8. Safety Evaluation of Hydrogels

According to ISO 10993-10 standards [77], SD rats were shaved on the back and treated with normal saline (control) and B-CGT twice daily. Skin condition was observed and scored on days 1, 2, 3, 4, 5, 6, and 7 post-application. The Primary Irritation Index (PII) was calculated as

(6) $$\mathrm{PII}=\frac{N}{n}$$

where N is the total score at each time point and n is the number of animals. A lower PII indicates lower irritation. In a further experiment, the shaved areas on the rats’ backs were slightly scratched to induce minor bleeding, washed with the control, and treated with hydrogel twice daily. Skin condition was scored and photographed at 1, 12, 24, and 48 h post-application. Scoring criteria are shown in Table 12 and Table 13.

### 3.9. Wound Healing Experimental Study

In this experiment, SD rats were selected and purchased from Hunan Jiatai Experimental Animal Co., Ltd. All animals were housed in SPF-level facilities with a 12 h light/dark cycle (Animal Ethics Approval No.20231228001). The 24 SD rats, weighing between 180 and 220 g, were randomly divided into three groups using a random number method: the control group, the CGT group (drug-free hydrogel), and the B-CGT group (drug-loaded hydrogel). There were 8 rats per group, housed individually. The day before the experiment, the rats’ backs were shaved and cleaned with saline. On the day of modeling, anesthesia was induced with isoflurane for a few minutes and maintained with a mask. The surgical area was disinfected with iodine, and a 10 mm full-thickness skin wound was created using a biopsy punch. The wounds were evenly treated with the control, CGT, or B-CGT. Digital images of the wounds were taken on post-operative days 3, 7, and 13 and measured using ImageJ software V1.8.0, NIH, Bethesda, MD, USA). The healing rate (%) was calculated as follows:

(7) $$\mathrm{Healing}\;\mathrm{rate}\;\left(\%\right)=\left(\frac{A_{0}-A_{\left(3,7,13\right)}}{A_{0}}\right)\times 100\%$$

where A_0_ and A_(3,7,13)_ represent the unhealed area on days 0, 3, 7, and 13, respectively. Histological and immunofluorescence analyses were performed on regenerated skin samples collected on post-operative days 3, 7, and 13. The samples were fixed in 4% paraformaldehyde and embedded in paraffin for subsequent staining.

#### 3.9.1. Hematoxylin and Eosin (HE) Staining

Paraffin-embedded tissue sections were deparaffinized using an eco-friendly deparaffinization solution (2x for 20 min each), followed by washing with absolute ethanol (2x for 5 min each) and 75% ethanol (1x for 5 min). The sections were then washed with water. Pre-treatment involved placing the sections in a high-definition staining pre-treatment solution for 1 min. The sections were stained with hematoxylin for 3 min, washed with running water, differentiated, blued, and rewashed. Eosin staining was performed by dehydrating the sections in 95% ethanol for 1 min and staining in eosin for 15 s. The sections were dehydrated in absolute ethanol (3x for 2 min each) and butanol (2x for 2 min each) and finally cleared in xylene (2x for 2 min each) and mounted with neutral resin. A NIKON ECLIPSE E100 microscope (NIKON CORPORATION, Tokyo, Japan) was used for examination. Image-Pro Plus 6.0 (Media Cybernetics, Inc., Rockville, MD, USA) was used to collect images and to measure the thickness of the granulation tissue and epidermis.

#### 3.9.2. Masson’s Trichrome Staining

Paraffin-embedded sections were deparaffinized to water, as described in the HE staining protocol. The sections were stained using the Masson’s trichrome staining kit according to the manufacturer’s instructions. The sections were immersed in Masson A solution overnight, washed with running water, and then stained with a mixture of Masson B and C solutions for 1 min, followed by differentiation and washing. The sections were then stained in Masson D solution for 6 min, washed with water, and further stained in Masson E solution for 1 min. After removal, the sections were placed directly in Masson F solution for 2–30 s, washed with 1% acetic acid for differentiation, and dehydrated in absolute ethanol. The sections were then cleared in xylene for 5 min and mounted with neutral resin. Microscopic examination was performed, images were captured, and the analysis was conducted using Image J software.

#### 3.9.3. Immunofluorescence Analysis

For the immunofluorescence analysis, deparaffinization (eco-friendly solution) and hydration (absolute ethanol) were followed by antigen retrieval using EDTA (pH = 8.0), ensuring the sections did not dry. The skin tissue sections were treated with PBS (pH = 7.4) three times for 5 min each at room temperature, followed by 25 min of incubation in 3% hydrogen peroxide in the dark to block endogenous peroxidase. After treatment with PBS, the sections were blocked with 3% BSA for 30 min. The sections were incubated with CD31 antibody at 4 °C overnight. After washing with PBS, HRP-conjugated secondary antibody was added, and the sections were incubated at room temperature for 50 min, followed by washing with PBS. CY3 was added and incubated in the dark for 10 min, followed by TBST treatment and microwave heating. CD163 antibody was then added and incubated at 4 °C overnight, washed with PBS, and incubated with Alexa-Fluor-488-conjugated secondary antibody in the dark for 50 min. DAPI staining solution was added and incubated in the dark for 10 min. Finally, autofluorescence quencher was added and incubated for 5 min. Fluorescence images were taken with a Nikon Eclipse C1 fluorescence microscope (NIKON CORPORATION, Tokyo, Japan).

### 3.10. Statistical Analysis

All experimental results were derived from at least three independent experiments. Statistical analyses were performed using SPSS software 22.0, IBM, Chicago, IL, USA, and data were presented as mean ± standard deviation (Mean ± SD). Depending on the experimental design, *t*-tests or one/two-way ANOVA were used for data analysis. Graphpad Prism 9.0 (GraphPad Software, Inc., Boston, MA, USA) was used for plotting. *p*-value < 0.05 was considered statistically significant.

## 4. Conclusions

In this study, building upon existing formulations, we developed a semi-solid gel, B-CGT, by screening matrix materials and evaluating formulation processes. This was the first attempt to prepare a gel from the extract of *B. striata* containing glucosyloxybenzyl 2-isobutylmalates. The experimental results demonstrated that this hydrogel possesses excellent biocompatibility and suitable physical properties, such as porosity, biodegradability, and adjustable mechanical properties. These characteristics effectively address the mechanical property requirements of certain special wounds and modulate the inflammatory microenvironment of wounds without the need for additional therapeutic agents.

Moreover, the hydrogel’s good swelling performance not only maintains a moist wound environment but also absorbs exudate from wound tissues, effectively preventing secondary trauma and promoting timely wound cleaning. The antioxidant capacity of B-CGT hydrogel balances the oxidative stress response, thus managing the overproduction of free radicals resulting from enhanced cellular metabolic activities and inflammatory responses.

Additionally, B-CGT hydrogel effectively promotes the proliferation, adhesion, and migration of NIH/3T3 cells in vitro, indicating good cell compatibility. In vivo studies confirmed that B-CGT hydrogel promotes wound healing by modulating the inflammatory microenvironment. It also facilitates the formation of new blood vessels (CD31) and shows a significant positive area ratio (CD163) with appropriate collagen deposition. Safety evaluations, including in vivo irritation tests and in vitro hemocompatibility tests, demonstrated that B-CGT hydrogel does not cause tissue reactions and exhibits good blood compatibility, confirming the safety of B-CGT hydrogel. These findings further support its application value in wound treatment.

Future research will explore the clinical application potential of this hydrogel, especially in the management of complex or non-healing diabetic wounds. Additionally, modification and functionalization of the hydrogel will be a key focus to enhance its therapeutic effects and applicability. Through these studies, we aim to bring this novel material into practical use, providing more options and better outcomes for wound healing treatments.

## Figures and Tables

**Table 1:** Selection of hydrogel preparation methods.

Preparation Method	Matrix Name	Appearance	Bubble	Mobility	Stickiness
Method ①	Carbomer-940	White semi-solid	+	++	+
	Gelatin	Light yellow semi-solid	-	++	-
	Chitosan	Milky precipitate	/	++	/
Method ②	Carbomer-940	White semi-solid	++	++	+
	Gelatin	Light yellow semi-solid	-	++	-
	Chitosan	Milky precipitate	/	++	/
Method ③	Carbomer-940	White semi-solid	-	++	-
	Gelatin	Light yellow semi-solid	-	++	-
	Chitosan	Milky precipitate	/	++	/

Note: ‘/’ means no such feature; ‘-’ means poor (visible bubbles >20, difficult to flow, and the viscosity range is between 1 and 100 cP); ‘+’ means general (visible bubbles n ≤ 20, not easy to flow, and the viscosity range is between 100 and 700 cP); ‘++’ represents good (visible bubbles n ≤5, easy to flow, and the viscosity range is between 700 and 1100 cP).

**Table 2:** Screening results of the matrix dosage.

Matrix Name	Dosage/(g/100mL)	Character	Stickiness	Formability	Stability
Carbomer	0.3	White semi-solid	-	-	++
	0.6	White semi-solid	++	+	++
	0.9	White semi-solid	++	++	++
Gelatin	0.3	Light yellow semi-solid	-	-	-
	0.6	Light yellow semi-solid	+	-	-
	0.9	Light yellow semi-solid	+	+	-
Chitosan	0.3	Milky precipitate	-	-	-
	0.6	Milky precipitate	-	-	-
	0.9	Milky precipitate	-	-	-

Note: ‘-’ means poor (the viscosity range is between 1 and 100 cP; not forming; stratification); ‘+’ means general (the viscosity range is between 100 and 700 cP; forming; not easy to stratify); ‘++’ means good (the viscosity range is between 700 and 1100 cP; good stability; not stratified).

**Table 3:** Screening of glycerol dosage in CGT matrix.

Glycerol (%)	Appearance	Spreadability	Viscosity	Stability	Greasiness
0	Brownish-yellow semi-solid	++	+	+	-
5	Brownish-yellow semi-solid	++	+	+	+
10	Brownish-yellow semi-solid	++	+	+	+
15	Brownish-yellow semi-solid	++	+	+	++
20	Brownish-yellow semi-solid	+	+	+	++

Note: “-” indicates absence (poor spreadability and application; the viscosity range is between 1 and 100 cP; stratification; no greasiness); “+” indicates moderate (spreadable, not easy to apply; the viscosity range is between 100 and 700 cP; stable; moderate greasiness); “++” indicates good (easy to spread and apply; the viscosity range is between 700 and 1100 cP; stable; slightly greasy).

**Table 4:** Screening of pH regulator triethanolamine in CGT matrix.

Triethanolamine (mL)	Appearance	Spreadability	Viscosity	Stability	pH
0.2	Brownish-yellow semi-solid	++	-	++	4.84
0.3	Brownish-yellow semi-solid	++	+	++	4.95
0.4	Brownish-yellow semi-solid	++	+	++	5.20
0.5	Brownish-yellow semi-solid	++	+	++	5.36
0.6	Brownish-yellow semi-solid	++	+	++	5.62
0.7	Brownish-yellow semi-solid	++	+	++	6.17
0.8	Brownish-yellow semi-solid	++	+	++	6.22
0.9	Brownish-yellow semi-solid	++	+	++	6.59
1	Brownish-yellow semi-solid	++	+	++	6.80

Note: “-” indicates absence (poor spreadability and application; the viscosity range is between 1 and 100 cP; stratification; no greasiness); “+” indicates moderate (spreadable, not easy to apply; the viscosity range is between 100 and 700 cP; stable; moderate greasiness); “++” indicates good (easy to spread and apply; the viscosity range is between 700 and 1100 cP; stable; slightly greasy).

**Table 5:** Response surface test design and results.

Experiment Number	Carbopol 940 (g)	Glycerol (mL)	Triethanolamine (mL)	Score
1	0.3	10	0.75	28
2	0.6	10	0.5	33
3	0.9	10	0.75	30
4	0.3	5	0.5	27
5	0.6	10	0.5	33
6	0.6	15	0.75	32
7	0.6	10	0.5	34
8	0.6	15	0.25	27
9	0.6	10	0.5	33
10	0.3	15	0.5	29
11	0.6	5	0.75	25
12	0.6	10	0.5	34
13	0.9	15	0.5	33
14	0.6	5	0.25	22
15	0.3	10	0.25	22
16	0.9	10	0.25	24
17	0.9	5	0.5	26

**Table 6:** Variance analysis of hydrogel preparation process screening.

Response Source	Sum of Squares	df	Mean Square	*F*-Value	*p*-Value
Model	276.49	9	30.72	48.33	<0.0001
A	6.13	1	6.13	9.63	0.0172
B	55.13	1	55.13	86.71	<0.0001
C	50	1	50	78.65	<0.0001
AB	6.25	1	6.25	9.83	0.0165
AC	0	1	0	0	1
BC	1	1	1	1.57	0.25
A2	27.92	1	27.92	43.92	0.0003
B2	18.13	1	18.13	28.52	0.0011
C2	98.02	1	98.02	154.19	<0.0001
Lack of Fit	3.25	3	1.08	3.61	0.1234
R^2^: 0.9842					
Adjusted R^2^: 0.9638					

Note: R^2^: 0.9842; Adjusted R^2^: 0.9638 (*p* < 0.01 indicates high significance, *p* < 0.05 indicates significance, *p* < 0.1 indicates non-significance). A represents the percentage content of Carbopol 940, B represents the percentage content of glycerol, and C represents the percentage content of triethanolamine.

**Table 7:** Comparative analysis of various hydrogel systems and their properties in wound healing.

Hydrogel Type	Biocompatibility	Moisture Retention	Mechanical Strength	Wound Healing Efficacy
Alginate Hydrogel	Moderate	High	Low	Delayed due to acidification
Hyaluronic Acid Hydrogel	High	Very High	Low (Rapid Degradation)	Good (but frequent replacement needed)
B-CGT Hydrogel	Excellent	High	Moderate to High	Significantly better wound closure and collagen deposition

**Table 8:** Primary Irritation Index (PII) scores.

Group	1 d	2 d	3 d	4 d	5 d	6 d	7 d
Control	0	0	0	0	0	0	0
B-CGT	0.03	0	0	0	0	0	0

**Table 9:** Scratch test scores.

Group	1 h	12 h	24 h	48 h
Control	0	0	0	0
B-CGT	0	0	0	0

**Table 10:** Response surface design factor levels table.

Level	Factor		
	Carbomer-940/(g)	Glycerol/(mL)	Triethanolamine/(mL)
−1	0.3	5	0.25
0	0.6	10	0.5
1	0.9	15	0.75

**Table 11:** Scoring standard of hydrogel sensory index.

Index of Evaluation	Request	Scale of Marks	Score
External properties	At room temperature, the hydrogel is clear and transparent, and it does not dry out or liquefy	Clear and transparent, not dry or liquefied	7~10
		More clear or transparent, but not liquefied or dry	4~6
		Unclear, or appears dry or liquefied, fluid dynamics	0~3
Homogeneity	The surface is smooth, uniform, and delicate; no/a small amount of bubbles exist; no precipitation	The surface is smooth, uniform, and delicate; no/a small amount of bubbles exist; no precipitation	7~10
		The surface is smooth, delicate, and uniform; a small number of small bubbles exists; no precipitation	4~6
		The surface is not smooth, delicate, or uniform, and there are larger or more bubbles or precipitation	0~3
Spreadability	It is sticky, easy to apply, and comfortable on the skin surface after application	Good viscosity, easy to apply, comfortable skin surface after application	7~10
		Poor viscosity, slightly difficult to apply; after applying, the skin surface is comfortable	4~6
		Poor viscosity, difficult to apply; the skin surface is not comfortable after application	0~3
Centrifugal stability	It should be uniform without variation and without stratification	Uniform without variation and without stratification	8
		Stratified	3
		Distinct stratification	0

**Table 12:** Scoring standard of hydrogel skin irritation test.

Reaction Type		Rate Range
**Formation of erythema and eschar**	Erythema-free	0
	Very slight erythema (barely visible)	1
	Clear erythema	2
	Moderate erythema	3
	Moderate to severe erythema (purplish red) to eschar formation	4
**Edema**	No edema	0
	Very slight edema (barely visible)	1
	Clear edema (swelling, no more than the edge of the region)	2
	Moderate edema (swelling of about 1 mm)	3
	Severe edema (swelling more than 1 mm and beyond the contact area)	4

**Table 13:** Scoring criteria for hydrogel Primary Irritation Index (PII) reaction types.

Stimulation Index	Reaction Type
0.0~0.4	Slight
0.5~1.9	Mild
2.0~4.9	Moderate
5.0~8.0	Serious

## Data Availability

Data will be made available upon request.

## Abbreviations

ABTS, 2,2′-azinobis-(3-ethylbenzothiazoline-6-sulfonic acid); B-CGT, glucosyloxybenzyl 2-Isobutylmalates extract hydrogel containing carbomer, glycerol, and triethanolamine; BSA, bovine serum albumin; CCK-8, Cell Counting Kit-8; CGT, carbomer, glycerol, and triethanolamine (non-drug-loaded hydrogel); DAPI, 4′,6-diamidino-2-phenylindole; DPPH, 2,2-diphenyl-1-picrylhydrazyl; ECM, extracellular matrix; EDTA, Ethylenediaminetetraacetic Acid; FTIR, Fourier transform infrared spectroscopy; HE, hematoxylin–eosin; HR, hemolysis rate; PBS, phosphate-buffered saline; PII, Primary Irritation Index; PRP-512B, reversed-phase resin type used for extract preparation; SEM, Scanning Electron Microscopy; SPF, specific-pathogen-free; TCP, Tissue Culture Plate.

## References

[1] Childs D.R., Murthy A.S. (2017). Overview of Wound Healing and Management. Surg. Clin. N. Am..

[2] Matsuzaki K., Upton D. (2012). Wound treatment and pain management: A stressful time. Int. Wound J..

[3] Woo K.Y. (2013). Trends in Wound Management. Adv. Ski. Wound Care.

[4] Zarchi K., Latif S., Haugaard V., Hjalager I., Jemec G. (2014). Significant Differences in Nurses’ Knowledge of Basic Wound Management—Implications for Treatment. Acta Dermato-Venereologica.

[5] Nathoo R., Howe N., Cohen G. (2014). Skin substitutes: An overview of the key players in wound management. J. Clin. Aesthet. Dermatol..

[6] Heras K.L., Igartua M., Santos-Vizcaino E., Hernandez R.M. (2022). Cell-based dressings: A journey through chronic wound management. Mater. Sci. Eng. C.

[7] Ananda N., Ariawan D., Juniantito V. (2022). Effects of the Hydnophytum formicarum plant extract on collagen density, angiogenesis, wound length, and re-epithelialization in wound healing: Experimental study on rats. Dent. Med. Probl..

[8] Ezzat S.M., Choucry M.A., Kandil Z.A. (2015). Antibacterial, antioxidant, and topical anti-inflammatory activities of *Bergia ammannioides*: A wound-healing plant. Pharm. Biol..

[9] Liang J., Cui L., Li J., Guan S., Zhang K., Li J. (2021). *Aloe vera*: A Medicinal Plant Used in Skin Wound Healing. Tissue Eng. Part B Rev..

[10] Mssillou I., Bakour M., Slighoua M., Laaroussi H., Saghrouchni H., Amrati F.E.-Z., Lyoussi B., Derwich E. (2022). Investigation on wound healing effect of Mediterranean medicinal plants and some related phenolic compounds: A review. J. Ethnopharmacol..

[11] Romo-Rico J., Krishna S.M., Bazaka K., Golledge J., Jacob M.V. (2022). Potential of plant secondary metabolite-based polymers to enhance wound healing. Acta Biomater..

[12] He X., Wang X., Fang J., Zhao Z., Huang L., Guo H., Zheng X. (2017). Bletilla striata: Medicinal uses, phytochemistry and pharmacological activities. J. Ethnopharmacol..

[13] Xu D., Pan Y., Chen J. (2019). Chemical Constituents, Pharmacologic Properties, and Clinical Applications of Bletilla striata. Front. Pharmacol..

[14] Zhu Z., Liang T., Dai G., Zheng J., Dong J., Xia C., Duan B. (2023). Extraction, structural-activity relationships, bioactivities, and application prospects of Bletilla striata polysaccharides as ingredients for functional products: A review. Int. J. Biol. Macromol..

[15] Song Y., Zeng R., Hu L., Maffucci K.G., Ren X., Qu Y. (2017). In vivo wound healing and in vitro antioxidant activities of Bletilla striata phenolic extracts. Biomed. Pharmacother..

[16] He X., Liu L., Gu F., Huang R., Liu L., Nian Y., Zhang Y., Song C. (2024). Exploration of the anti-inflammatory, analgesic, and wound healing activities of Bletilla Striata polysaccharide. Int. J. Biol. Macromol..

[17] Si Y., Guo C., Xu X., Zhang K., Tan R., Lau K.-T., Hu J. (2022). Bioinspired Janus All-Natural Electrospinning Membranes with Directional Water Transport as Ecofriendly Dry Facial Masks. ACS Sustain. Chem. Eng..

[18] Fang Y.-K., Shang Z.-M., Sun G.-Q., Zhang M.-S., Wang G., Xu D.-L., Zhou Y., Sun C.-X., Xiao S.-J. (2022). Glucosyloxybenzyl 2-isobutylmalates and phenolic glycosides from the flowers of Bletilla striata. Fitoterapia.

[19] Jiang S., Wang M., Jiang L., Xie Q., Yuan H., Yang Y., Zafar S., Liu Y., Jian Y., Li B. (2021). The medicinal uses of the genus Bletilla in traditional Chinese medicine: A phytochemical and pharmacological review. J. Ethnopharmacol..

[20] Lee C.-L., Jhan Y.-L., Chiang H.-M., Chen C.-J. (2023). Bioactive phytochemicals from the tubers of *Bletilla striata* Rchb.f. Nat. Prod. Res..

[21] Zhou M., Yuan F., Ruan H., Li J., Huang J., Liu S., Huang T., Zhang Y., Liang Q. (2022). HPLC-PDA-Guided isolation of glucosyloxybenzyl 2-isobutylmalates from the pseudobulbs of Bletilla striata with neuroprotective and antimicrobial activities. Phytochemistry.

[22] Mu K., Liu Y., Liu G., Ran F., Zhou L., Wu Y., Peng L., Shao M., Li C., Zhang Y. (2023). A review of hemostatic chemical components and their mechanisms in traditional Chinese medicine and ethnic medicine. J. Ethnopharmacol..

[23] Liu G., Mu K., Ran F., Liu J., Zhou L., Peng L., Feng G., Liu Y., Wei F., Zhu L. (2024). The hemostatic activity and Mechanistic roles of glucosyloxybenzyl 2-isobutylmalate extract (BSCE) from Bletilla striata (Thunb.) Rchb.f. in Inhibiting pulmonary hemorrhage. Heliyon.

[24] Li Z., Yu D. (2023). Controlled ibuprofen release from Pickering emulsions stabilized by pH-responsive cellulose-based nanofibrils. Int. J. Biol. Macromol..

[25] Wang Z., Ye Q., Yu S., Akhavan B. (2023). Poly Ethylene Glycol (PEG)-Based Hydrogels for Drug Delivery in Cancer Therapy: A Comprehensive Review. Adv. Healthc. Mater..

[26] Guo W., Cao D., Rao W., Sun T., Wei Y., Wang Y., Yu L., Ding J. (2023). Achieving Long-Acting Local Analgesia Using an Intelligent Hydrogel Encapsulated with Drug and pH Regulator. ACS Appl. Mater. Interfaces.

[27] George B., Janis J.E., Attinger C.E. (2006). Wound Healing: An Overview. Plast. Reconstr. Surg..

[28] Park H., Copeland C., Henry S., Barbul A. (2010). Complex Wounds and Their Management. Surg. Clin. N. Am..

[29] Bano I., Arshad M., Yasin T., Ghauri M.A., Younus M. (2017). Chitosan: A potential biopolymer for wound management. Int. J. Biol. Macromol..

[30] Lindholm C., Searle R. (2016). Wound management for the 21st century: Combining effectiveness and efficiency. Int. Wound J..

[31] Yuan N., Shao K., Huang S., Chen C. (2023). Chitosan, alginate, hyaluronic acid and other novel multifunctional hydrogel dressings for wound healing: A review. Int. J. Biol. Macromol..

[32] Lv H., Wu B., Song J., Wu W., Cai W., Xu J. (2021). Hydrogel, a novel therapeutic and delivery strategy, in the treatment of intrauterine adhesions. J. Mater. Chem. B.

[33] Zhang A., Liu Y., Qin D., Sun M., Wang T., Chen X. (2020). Research status of self-healing hydrogel for wound management: A review. Int. J. Biol. Macromol..

[34] Wu Y., Wang J., Li L., Fei X., Xu L., Wang Y., Tian J., Li Y. (2020). A novel hydrogel with self-healing property and bactericidal activity. J. Colloid Interface Sci..

[35] Budai L., Budai M., Pápay Z.E.F., Szalkai P., Niczinger N.A., Kijima S., Sugibayashi K., Antal I., Kállai-Szabó N. (2023). Viscoelasticity of Liposomal Dispersions. Nanomaterials.

[36] Rogina A., Ressler A., Matić I., Ferrer G.G., Marijanović I., Ivanković M., Ivanković H. (2017). Cellular hydrogels based on pH-responsive chitosan-hydroxyapatite system. Carbohydr. Polym..

[37] Gupta R., Kumar A. (2008). RETRACTED: Molecular imprinting in sol–gel matrix. Biotechnol. Adv..

[38] Rachmawati P. (2023). Basics: A Review: In Situ Gel Dosage Forms for Herbal Medicine Delivery. Int. J. Pharm. Compd..

[39] Ali S.M., Yosipovitch G. (2013). Skin pH: From Basic SciencE to Basic Skin Care. Acta Derm. Venereol..

[40] Proksch E. (2018). pH in nature, humans and skin. J. Dermatol..

[41] Schmid-Wendtner M.-H., Korting H. (2006). The pH of the Skin Surface and Its Impact on the Barrier Function. Ski. Pharmacol. Physiol..

[42] Chinese Pharmacopoeia Commission (2020). Pharmacopoeia of the People’s Republic of China.

[43] Tang R., Zhang Y., Zhang Y., Yu Z. (2016). Synthesis and characterization of chitosan based dye containing quaternary ammonium group. Carbohydr. Polym..

[44] Chen K.-Y., Zeng S.-Y. (2018). Fabrication of Quaternized Chitosan Nanoparticles Using Tripolyphosphate/Genipin Dual Cross-Linkers as a Protein Delivery System. Polymers.

[45] Nhung L.T.T., Kim I.Y., Yoon Y.S. (2020). Quaternized Chitosan-Based Anion Exchange Membrane Composited with Quaternized Poly(vinylbenzyl chloride)/Polysulfone Blend. Polymers.

[46] Bernhardt D.C., Pérez C.D., Fissore E.N., De’nobili M.D., Rojas A.M. (2017). Pectin-based composite film: Effect of corn husk fiber concentration on their properties. Carbohydr. Polym..

[47] Ahmed E.M. (2015). Hydrogel: Preparation, characterization, and applications: A review. J. Adv. Res..

[48] Catoira M.C., Fusaro L., Di Francesco D., Ramella M., Boccafoschi F. (2019). Overview of natural hydrogels for regenerative medicine applications. J. Mater. Sci. Mater. Med..

[49] Boateng J.S., Matthews K.H., Stevens H.N., Eccleston G.M. (2008). Wound Healing Dressings and Drug Delivery Systems: A Review. J. Pharm. Sci..

[50] Hendi A., Hassan M.U., Elsherif M., Alqattan B., Park S., Yetisen A.K., Butt H. (2020). Healthcare Applications of pH-Sensitive Hydrogel-Based Devices: A Review. Int. J. Nanomed..

[51] Das I.J., Bal T. (2024). Exploring carrageenan: From seaweed to biomedicine—A comprehensive review. Int. J. Biol. Macromol..

[52] Sanjanwala D., Londhe V., Trivedi R., Bonde S., Sawarkar S., Kale V., Patravale V. (2024). Polysaccharide-based hydrogels for medical devices, implants and tissue engineering: A review. Int. J. Biol. Macromol..

[53] Hynes R.O. (2002). Integrins. Cell.

[54] Martin P. (1997). Wound Healing—Aiming for Perfect Skin Regeneration. Science.

[55] (2009). Biological Evaluation of Medical Devices—Part 5: Tests for In Vitro Cytotoxicity.

[56] (2017). Biological Evaluation of Medical Devices—Part 4: Selection of Tests for Interactions with Blood.

[57] (2017). Insights into In Vitro Hemocompatibility Testing According to ISO 10993-4.

[58] Zhang M., Zhao X. (2020). Alginate hydrogel dressings for advanced wound management. Int. J. Biol. Macromol..

[59] Graça M.F.P., Miguel S.P., Cabral C.S.D., Correia I.J. (2020). Hyaluronic acid—Based wound dressings: A review. Carbohydr. Polym..

[60] Burdick J.A., Prestwich G.D. (2011). Hyaluronic acid hydrogels for biomedical applications. Adv. Mater..

[61] (2010). Biological Evaluation of Medical Devices—Part 10: Tests for Irritation and Skin Sensitization.

[62] Rousselle P., Braye F., Dayan G. (2018). Re-epithelialization of adult skin wounds: Cellular mechanisms and therapeutic strategies. Adv. Drug Deliv. Rev..

[63] Gawronska-Kozak B., Grabowska A., Kur-Piotrowska A., Kopcewicz M. (2016). Foxn1 Transcription Factor Regulates Wound Healing of Skin through Promoting Epithelial-Mesenchymal Transition. PLoS ONE.

[64] Eming S.A., Wynn T.A., Martin P. (2017). Inflammation and metabolism in tissue repair and regeneration. Science.

[65] Reinke J., Sorg H. (2012). Wound Repair and Regeneration. Eur. Surg. Res..

[66] Barrientos S., Stojadinovic O., Golinko M.S., Brem H., Tomic-Canic M. (2008). PERSPECTIVE ARTICLE: Growth factors and cytokines in wound healing. Wound Repair. Regen.

[67] Huang C., Dong L., Zhao B., Lu Y., Huang S., Yuan Z., Luo G., Xu Y., Qian W. (2022). Anti-inflammatory hydrogel dressings and skin wound healing. Clin. Transl. Med..

[68] Peña O.A., Martin P. (2024). Cellular and molecular mechanisms of skin wound healing. Nat. Rev. Mol. Cell Biol..

[69] Ehrlich H.P. (1988). Wound closure: Evidence of cooperation between fibroblasts and collagen matrix. Eye.

[70] Gardeazabal L., Izeta A. (2024). Elastin and collagen fibres in cutaneous wound healing. Exp. Dermatol..

[71] Kuivaniemi H., Tromp G. (2019). Type III collagen (COL3A1): Gene and protein structure, tissue distribution, and associated diseases. Gene.

[72] Ribatti D., Tamma R. (2019). Giulio Gabbiani and the discovery of myofibroblasts. Inflamm. Res..

[73] Smith P.C., Martínez C., Martínez J., McCulloch C.A. (2019). Role of Fibroblast Populations in Periodontal Wound Healing and Tissue Remodeling. Front. Physiol..

[74] des Jardins-Park H.E., Foster D.S., Longaker M.T. (2018). Fibroblasts and wound healing: An update. Regen. Med..

[75] Kalluri R. (2016). The biology and function of fibroblasts in cancer. Nat. Rev. Cancer.

[76] Liu G., Liu J.-m., Mu K.-l., Liu Y.-c., Sun Q.-w., Zhang Y.-q. (2023). Study on the application of Glucosyloxybenzyl 2-Isobutylmalates reference extract in the quality control of Bletilla striata. Chin. Herb. Med..

[77] (2020). Biological Evaluation of Medical Devices—Part 10: Tests for Irritation and Skin Sensitization.

